# Supplementary material for: Semi-field evaluation of human landing catches versus human double net trap for estimating human biting rate of Anopheles minimus and Anopheles harrisoni in Thailand
Source: PeerJ. 2022 Sep 8;10:e13865. doi: 10.7717/peerj.13865 (PMC9464434; doi:10.7717/peerj.13865)
Supplement: Supplemental Information 1 [file peerj-10-13865-s001.docx]

| Collection date | Number of *An. harrisoni* (field) mosquitoes released | | Number of *landing An. harrisoni* (field) recaptured | | Number of resting *An. harrisoni* (field) recaptured | | Total recaptured/night | Recaptured (%) |
| --- | --- | --- | --- | --- | --- | --- | --- | --- |
|  | HDNT | HLC | HDNT | HLC | HDNT | HLC |  |  |
| 1-Mar-2021 | 50 | 50 | 12 | 11 | 16 | 33 | 72 | 72 |
| 2-Mar-2021 | 50 | 50 | 4 | 17 | 27 | 18 | 66 | 66 |
| 3-Mar-2021 | 50 | 50 | 17 | 20 | 27 | 30 | 94 | 94 |
| 6-Mar-2021 | 50 | 50 | 6 | 23 | 25 | 22 | 76 | 76 |
| 7-Mar-2021 | 50 | 50 | 11 | 19 | 30 | 23 | 83 | 83 |
| 8-Mar-2021 | 50 | 50 | 3 | 23 | 39 | 27 | 92 | 92 |
| 21-Mar-2021 | 50 | 50 | 32 | 16 | 14 | 26 | 88 | 88 |
| 22-Mar-2021 | 50 | 50 | 15 | 26 | 33 | 18 | 92 | 92 |
| 23-Mar-2021 | 50 | 50 | 11 | 13 | 29 | 28 | 81 | 81 |
| 27-Mar-2021 | 50 | 50 | 6 | 16 | 36 | 26 | 84 | 84 |
| 28-Mar-2021 | 50 | 50 | 16 | 10 | 28 | 35 | 89 | 89 |
| 30-Mar-2021 | 50 | 50 | 10 | 28 | 33 | 18 | 89 | 89 |
| 4-Apr-2021 | 50 | 50 | 20 | 11 | 24 | 32 | 87 | 87 |
| 10-Apr-2021 | 50 | 50 | 10 | 21 | 34 | 22 | 87 | 87 |
| 11-Apr-2021 | 50 | 50 | 15 | 13 | 28 | 30 | 86 | 86 |
| 13-Apr-2021 | 50 | 50 | 13 | 20 | 30 | 24 | 87 | 87 |
| 18-Apr-2021 | 50 | 50 | 12 | 22 | 31 | 22 | 87 | 87 |
| 20-Apr-2021 | 50 | 50 | 10 | 21 | 33 | 23 | 87 | 87 |
| 24-Apr-2021 | 50 | 50 | 19 | 25 | 23 | 19 | 86 | 86 |
| 25-Apr-2021 | 50 | 50 | 28 | 28 | 18 | 16 | 90 | 90 |
| 26-Apr-2021 | 50 | 50 | 29 | 24 | 16 | 20 | 89 | 89 |
| 27-Apr-2021 | 50 | 50 | 28 | 24 | 16 | 19 | 87 | 87 |
| 28-Apr-2021 | 50 | 50 | 29 | 19 | 16 | 26 | 90 | 90 |
| 29-Apr-2021 | 50 | 50 | 17 | 20 | 26 | 23 | 86 | 86 |
| 30-Apr-2021 | 50 | 50 | 23 | 20 | 23 | 23 | 89 | 89 |
| 3-May-2021 | 50 | 50 | 8 | 31 | 35 | 13 | 87 | 87 |
| 5-May-2021 | 50 | 50 | 22 | 22 | 21 | 21 | 86 | 86 |
| 6-May-2021 | 50 | 50 | 21 | 39 | 25 | 5 | 90 | 90 |
| 9-May-2021 | 50 | 50 | 7 | 35 | 36 | 8 | 86 | 86 |
| 10-May-2021 | 50 | 50 | 11 | 16 | 32 | 28 | 87 | 87 |
| **Total recaptured** | **1500** | **1500** | **465** | **633** | **804** | **678** | **2580** | **86** |
| **Percent of recaptured (%)** | |  | **15.5** | **21.1** | **26.8** | **22.6** |  |  |
