# Supplementary material for: Semi-field evaluation of human landing catches versus human double net trap for estimating human biting rate of Anopheles minimus and Anopheles harrisoni in Thailand
Source: PeerJ. 2022 Sep 8;10:e13865. doi: 10.7717/peerj.13865 (PMC9464434; doi:10.7717/peerj.13865)
Supplement: Supplemental Information 2 [file peerj-10-13865-s002.docx]

| Collection date | Number of *An. minimus* released | | Number of landing *An. minimus* recaptured | | Number of resting *An. minimus* recaptured | | Total recaptured/night | Recaptured (%) |
| --- | --- | --- | --- | --- | --- | --- | --- | --- |
|  | HDNT | HLC | HDNT | HLC | HDNT | HLC |  |  |
| 4-May-2021 | 50 | 50 | 36 | 47 | 10 | 3 | 96 | 96 |
| 7-May-2021 | 50 | 50 | 34 | 48 | 9 | 2 | 93 | 93 |
| 8-May-2021 | 50 | 50 | 42 | 45 | 3 | 1 | 91 | 91 |
| 11-May-2021 | 50 | 50 | 34 | 49 | 11 | 0 | 94 | 94 |
| 12-May-2021 | 50 | 50 | 38 | 47 | 6 | 2 | 93 | 93 |
| 13-May-2021 | 50 | 50 | 21 | 46 | 25 | 2 | 94 | 94 |
| 15-May-2021 | 50 | 50 | 36 | 48 | 11 | 1 | 96 | 96 |
| 16-May-2021 | 50 | 50 | 45 | 48 | 2 | 0 | 95 | 95 |
| 17-May-2021 | 50 | 50 | 49 | 43 | 0 | 1 | 93 | 93 |
| 18-May-2021 | 50 | 50 | 35 | 49 | 9 | 1 | 94 | 94 |
| 19-May-2021 | 50 | 50 | 44 | 47 | 1 | 0 | 92 | 92 |
| 20-May-2021 | 50 | 50 | 27 | 48 | 16 | 0 | 91 | 91 |
| 21-May-2021 | 50 | 50 | 43 | 43 | 2 | 0 | 88 | 88 |
| 22-May-2021 | 50 | 50 | 23 | 48 | 21 | 0 | 92 | 92 |
| 23-May-2021 | 50 | 50 | 44 | 40 | 6 | 6 | 96 | 96 |
| 24-May-2021 | 50 | 50 | 42 | 47 | 3 | 0 | 92 | 92 |
| 25-May-2021 | 50 | 50 | 43 | 47 | 4 | 0 | 94 | 94 |
| 26-May-2021 | 50 | 50 | 21 | 47 | 25 | 0 | 93 | 93 |
| 27-May-2021 | 50 | 50 | 45 | 42 | 1 | 3 | 91 | 91 |
| 28-May-2021 | 50 | 50 | 30 | 48 | 18 | 0 | 96 | 96 |
| 29-May-2021 | 50 | 50 | 45 | 47 | 4 | 0 | 96 | 96 |
| 30-May-2021 | 50 | 50 | 36 | 46 | 13 | 2 | 97 | 97 |
| 31-May-2021 | 50 | 50 | 49 | 48 | 0 | 0 | 97 | 97 |
| 1-Jun-2021 | 50 | 50 | 46 | 50 | 0 | 0 | 96 | 96 |
| 2-Jun-2021 | 50 | 50 | 48 | 49 | 1 | 0 | 98 | 98 |
| 3-Jun-2021 | 50 | 50 | 46 | 49 | 4 | 0 | 99 | 99 |
| 4-Jun-2021 | 50 | 50 | 40 | 49 | 8 | 0 | 97 | 97 |
| 5-Jun-2021 | 50 | 50 | 30 | 49 | 19 | 0 | 98 | 98 |
| 6-Jun-2021 | 50 | 50 | 43 | 48 | 6 | 0 | 97 | 97 |
| 7-Jun-2021 | 50 | 50 | 21 | 48 | 25 | 2 | 96 | 96 |
| **Total recaptured** | **1500** | **1500** | **1136** | **1410** | **263** | **26** | **2835** | **94.5** |
| **Percent of recaptured (%)** | |  | **37.87** | **47** | **8.77** | **0.87** |  |  |
