# Supplementary material for: Semi-field evaluation of human landing catches versus human double net trap for estimating human biting rate of Anopheles minimus and Anopheles harrisoni in Thailand
Source: PeerJ. 2022 Sep 8;10:e13865. doi: 10.7717/peerj.13865 (PMC9464434; doi:10.7717/peerj.13865)
Supplement: Supplemental Information 3 — *Significant at p < 0.05. [file peerj-10-13865-s003.docx]

| Mosquito species | Preferences of recapture | Traps | Spearman's correlation coefficient | | Density-dependence  (Simple linear regression) | | |
| --- | --- | --- | --- | --- | --- | --- | --- |
|  |  |  | R | *p*-value | R^2^ | t | *p*-value |
| *An. harrisoni* | Landing | HDNT-HLC | −0.020 | 0.916 | 0.237 | 2.949 | 0.006* |
|  | Resting | HDNT-HLC | −0.081 | 0.672 | 0.089 | −1.657 | 0.109 |
| *An. minimus* | Landing | HDNT-HLC | −0.090 | 0.636 | 0.866 | 13.433 | <0.001* |
|  | Resting | HDNT-HLC | 0.169 | 0.372 | 0.026 | −0.870 | 0.392 |
