# Supplementary material for: Semi-field evaluation of human landing catches versus human double net trap for estimating human biting rate of Anopheles minimus and Anopheles harrisoni in Thailand
Source: PeerJ. 2022 Sep 8;10:e13865. doi: 10.7717/peerj.13865 (PMC9464434; doi:10.7717/peerj.13865)
Supplement: Supplemental Information 4 — a Negative mean log ratio indicates that the efficiency of HDNT was lower than HLC. [file peerj-10-13865-s004.docx]

| Mosquito species | Preferences of recapture | Traps-HLC | Mean log ratio ^a^ | SEM | GMR | 95% CI | |
| --- | --- | --- | --- | --- | --- | --- | --- |
|  |  |  |  |  |  | Lower bound | Upper bound |
| *An. harrisoni* | Landing | HDNT | −0.240 | 0.501 | 0.000 | −0.341 | −0.137 |
|  | Resting | HDNT | 0.080 | 0.039 | 0.000 | 0.0005 | 0.159 |
| *An. minimus* | Landing | HDNT | −0.182 | 0.021 | 0.145 | −0.226 | −0.139 |
|  | Resting | HDNT | 0.0625 | 0.085 | 0.000 | 0.451 | 0.800 |
