# Supplementary material for: Semi-field evaluation of human landing catches versus human double net trap for estimating human biting rate of Anopheles minimus and Anopheles harrisoni in Thailand
Source: PeerJ. 2022 Sep 8;10:e13865. doi: 10.7717/peerj.13865 (PMC9464434; doi:10.7717/peerj.13865)
Supplement: Supplemental Information 6 — ∗Significant at p < 0.05. [file peerj-10-13865-s006.docx]

| Mosquito species | Weather | Traps | Spearman's correlation coefficient | |
| --- | --- | --- | --- | --- |
|  |  |  | R | *p*-value |
| *An. harrisoni* | Temperature | HDNT-HLC | 0.085 | 0.657 |
|  | Relative humidity | HDNT-HLC | 0.499 | 0.005* |
| *An. minimus* | Temperature | HDNT-HLC | 0.133 | 0.482 |
|  | Relative humidity | HDNT-HLC | 0.161 | 0.394 |
