# Supplementary material for: Semi-field evaluation of human landing catches versus human double net trap for estimating human biting rate of Anopheles minimus and Anopheles harrisoni in Thailand
Source: PeerJ. 2022 Sep 8;10:e13865. doi: 10.7717/peerj.13865 (PMC9464434; doi:10.7717/peerj.13865)
Supplement: Supplemental Information 7 [file peerj-10-13865-s007.pdf]

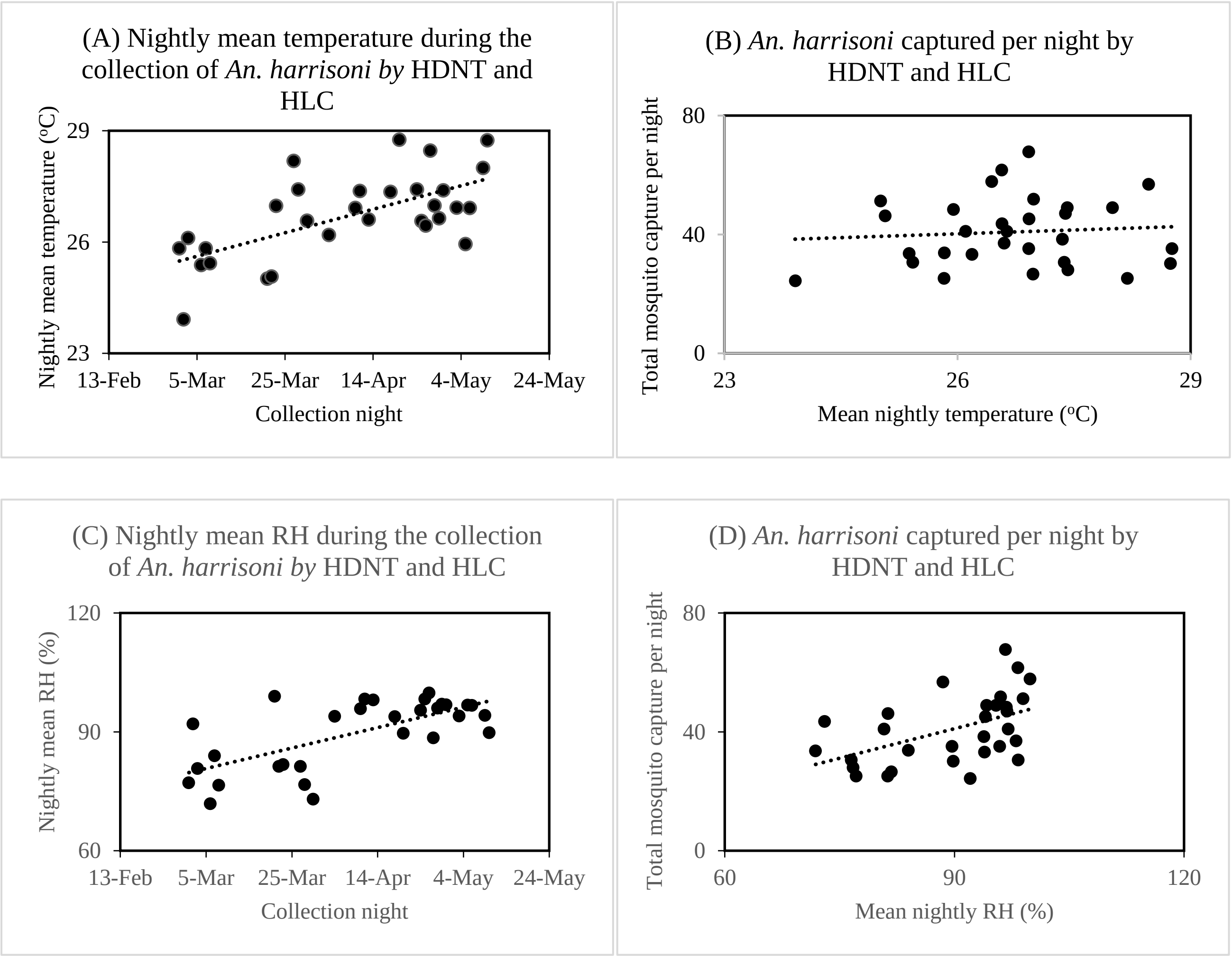
(A) Nightly mean temperature during the collection of An. harrisoni by HDNT and HLC
Nightly mean temperature (ºC)
29
26
23
13-Feb
5-Mar
25-Mar
14-Apr
4-May
24-May
Collection night
(B) An. harrisoni captured per night by HDNT and HLC
Total mosquito capture per night
80
40
0
23
26
29
Mean nightly temperature (ºC)
(C) Nightly mean RH during the collection of An. harrisoni by HDNT and HLC
Nightly mean RH (%)
120
90
60
13-Feb
5-Mar
25-Mar
14-Apr
4-May
24-May
Collection night
(D) An. harrisoni captured per night by HDNT and HLC
Total mosquito capture per night
80
40
0
60
90
120
Mean nightly RH (%)
