# Supplementary material for: Semi-field evaluation of human landing catches versus human double net trap for estimating human biting rate of Anopheles minimus and Anopheles harrisoni in Thailand
Source: PeerJ. 2022 Sep 8;10:e13865. doi: 10.7717/peerj.13865 (PMC9464434; doi:10.7717/peerj.13865)
Supplement: Supplemental Information 8 [file peerj-10-13865-s008.pdf]

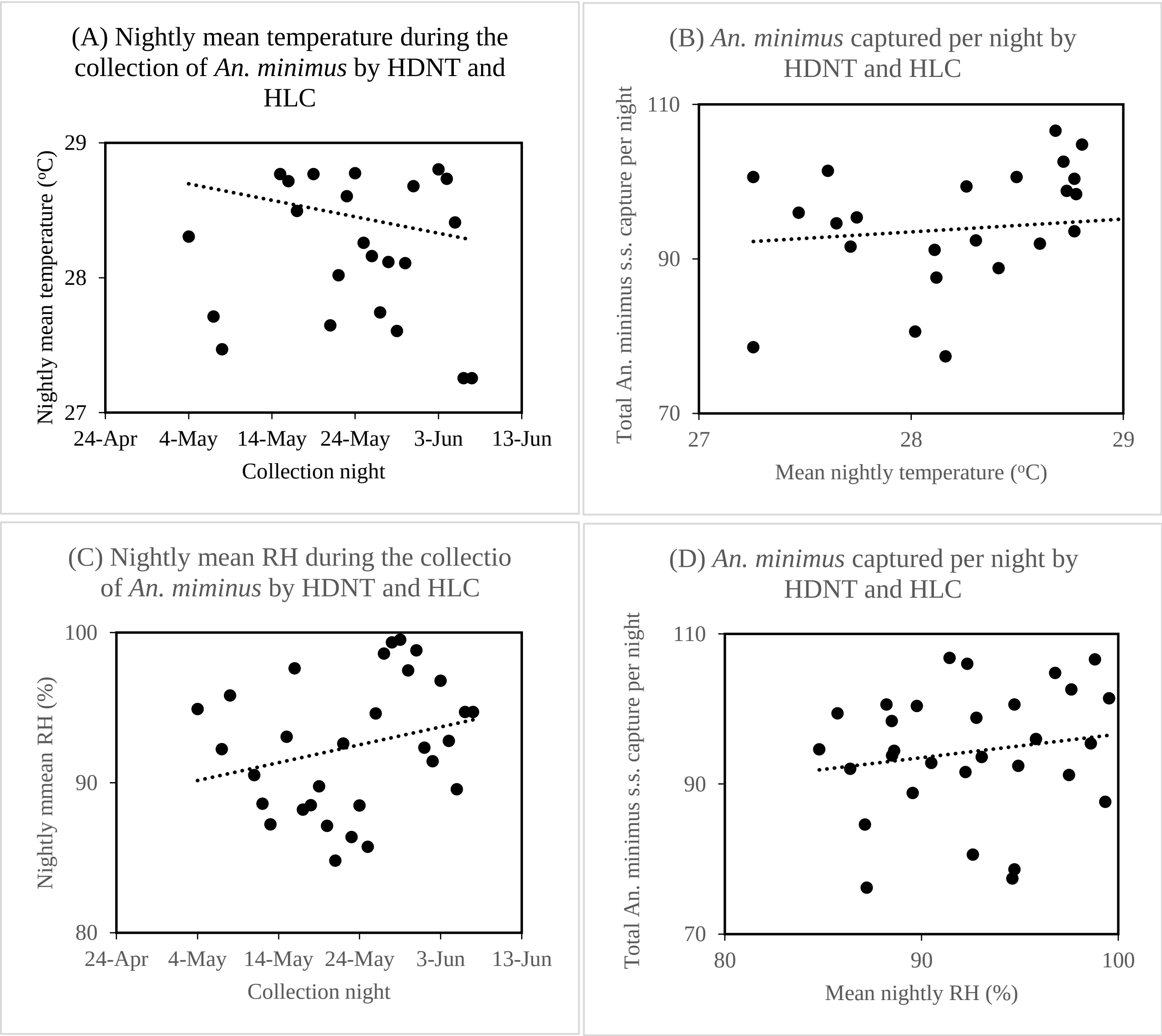

(A) Nightly mean temperature during the collection of *An. minimus* by HDNT and HLC
Nightly mean temperature (ºC)
29
28
27
24-Apr
4-May
14-May
24-May
3-Jun
13-Jun
Collection night
(B) *An. minimus* captured per night by HDNT and HLC
Total An. minimus s.s. capture per night
110
90
70
27
28
29
Mean nightly temperature (ºC)
(C) Nightly mean RH during the collectio of *An. miminus* by HDNT and HLC
Nightly mmean RH (%)
100
90
80
24-Apr
4-May
14-May
24-May
3-Jun
13-Jun
Collection night
(D) *An. minimus* captured per night by HDNT and HLC
Total An. minimus s.s. capture per night
110
90
70
80
90
100
Mean nightly RH (%)
